# Cytoplasmic Lipases—A Novel Class of Fungal Defense Proteins Against Nematodes

**DOI:** 10.3389/ffunb.2021.696972

**Published:** 2021-07-01

**Authors:** Annageldi Tayyrov, Chunyue Wei, Céline Fetz, Aleksandr Goryachkin, Philipp Schächle, Laura Nyström, Markus Künzler

**Affiliations:** ^1^Department of Biology, Institute of Microbiology, ETH Zürich, Zurich, Switzerland; ^2^Department of Health Sciences and Technology, Institute of Food, Nutrition and Health, ETH Zürich, Zurich, Switzerland

**Keywords:** *Coprinopsis cinerea*, fungal toxins, toxic enzymes, inducible defense, nematotoxicity, esterase, active site residues

## Abstract

Fungi are an attractive food source for predators such as fungivorous nematodes. Several fungal defense proteins and their protective mechanisms against nematodes have been described. Many of these proteins are lectins which are stored in the cytoplasm of the fungal cells and bind to specific glycan epitopes in the digestive tract of the nematode upon ingestion. Here, we studied two novel nematotoxic proteins with lipase domains from the model mushroom *Coprinopsis cinerea*. These cytoplasmically localized proteins were found to be induced in the vegetative mycelium of *C. cinerea* upon challenge with fungivorous nematode *Aphelenchus avenae*. The proteins showed nematotoxicity when heterologously expressed in *E. coli* and fed to several bacterivorous nematodes. Site-specific mutagenesis of predicted catalytic residues eliminated the *in-vitro* lipase activity of the proteins and significantly reduced their nematotoxicity, indicating the importance of the lipase activity for the nematotoxicity of these proteins. Our results suggest that cytoplasmic lipases constitute a novel class of fungal defense proteins against predatory nematodes. These findings improve our understanding of fungal defense mechanisms against predators and may find applications in the control of parasitic nematodes in agriculture and medicine.

## Introduction

Due to their lack of mobility and high nutrient content, fungi are an ideal food source for many predatory organisms (Ruess and Lussenhop, 2005; Boddy and Jones, 2008; Doll et al., 2013). In order to protect themselves against predation, fungi have evolved various defense mechanisms (Kunzler, 2018). In addition to physical defense (Gomez and Nosanchuk, 2003; Latge, 2007) or chemical defense by secondary metabolites (Rohlfs and Churchill, 2011; Spiteller, 2015; Keller, 2019), fungi rely on chemical defense mediated by proteins and peptides (Sabotic et al., 2016; Tayyrov et al., 2018). Several fungal defense proteins, biotin-binding protein(Bleuler-Martinez et al., 2012),including lectins (Bleuler-Martinez et al., 2011), protease inhibitors (Renko et al., 2010),pore-forming proteins (Mancheno et al., 2010), and ribotoxins (Lacadena et al., 2007; Tayyrov et al., 2019a) have been characterized. Considering the high diversity of fungal predators and the specificity of many defense effectors with regard to target organisms, the diversity of fungal defense proteins must be huge. It has been shown that genes encoding for fungal defense proteins can be identified on the basis of their expression upon challenge of a fungus with its antagonists (Schroeckh et al., 2009; Caballero Ortiz et al., 2013; Mathioni et al., 2013; Spraker et al., 2018; Tauber et al., 2018; Kombrink et al., 2019). Hence, we previously conducted an RNA seq-based genome-wide gene expression analysis of the model mushroom *Coprinopsis cinerea* challenged by the fungivorous nematode *Aphelenchus avenae* (Tayyrov et al., 2019b).

Here, we report on the characterization of two highly induced *C. cinerea* genes encoding for P452912 and P430758 (JGI MycoCosm ProteinIDs). The two genes showed expression dynamics that were similar to previously characterized nematotoxic proteins (CGL2, CCTX2 and P450139) (Bleuler-Martinez et al., 2011; Plaza et al., 2015; Tayyrov et al., 2019b). The encoded proteins contain putative lipase domains and showed toxicity toward the bacterivorous nematodes. In order to reflect the likely nature of their biological activity, we renamed P452912 and P430758 to Coprinopsis Lipase Toxin-1 (CLT1) and Coprinopsis Lipase Toxin-2 (CLT2), respectively. Site-specific mutagenesis of predicted catalytic residues significantly reduced the toxicity and abolished *in-vitro* lipase activity of the proteins, suggesting that the nematotoxicity of the toxins is dependent on a functional lipase domain.

The two main functions of lipids in biology are their structural role in biological membranes (Hunte and Richers, 2008) and their role in energy storage (Murphy, 2001). In addition, lipids have a multitude of other functions in several essential biological processes such as cellular signaling (Berridge and Irvine, 1989), cellular organization (Simons and Ikonen, 2000) and membrane trafficking (Haucke and Di Paolo, 2007). This multi-functionality and essentiality of lipids make them an ideal target for pathogens and competitors (Van Der Meer-Janssen et al., 2010). Accordingly, both bacterial and fungal pathogens employ lipases as virulence factors (Saising et al., 2012; Park et al., 2013; Singh et al., 2014; Staniszewska, 2020). In fact, there are many secreted bacterial toxins that contain lipase domains (Schmiel and Miller, 1999; Russell et al., 2013). The type III secretory toxin, ExoU, as a major virulence factor of *Pseudomonas aeruginosa* (Sato et al., 2003; Sato and Frank, 2004) and the T6SS effector of *Vibrio cholera*, the lipase TseL (Dong et al., 2013), are well-studied examples of lipase toxins being used against its eukaryotic host cells and in self-protection against bacterial competitors, respectively. Secreted lipases and proteases are used by various fungal pathogens, including *Candida* sp., *Malassezia* sp., and *Cryptococcus* sp., to invade host tissues (Schaller et al., 2005; Jones et al., 2007; Juntachai et al., 2009; Toth et al., 2017). Protozoan parasites are also known to target the lipidome of their hosts to establish the parasitic stage (Rub et al., 2013). Furthermore, venoms of certain insects and reptiles are known to contain lipases (Aloulou et al., 2012).

Our results suggest that proteins with functional lipase domains represent a novel class of fungal defense proteins against nematode predators.

## Materials and Methods

### Strains and Cultivation Conditions

*E. coli* DH5α was used for cloning and plasmid amplification. Heterologous protein expression was performed in *E. coli* BL21 (DE3). *Coprinopsis cinerea* AmutBmut strain was maintained on YMG [0.4% (w/v) yeast extract, 1% (w/v) malt extract, 0.4% (w/v) glucose, 1.5% (w/v) agar] at 37°C in a dark chamber. *Aphelenchus avenae* was propagated at 20°C on *Botrytis cinerea* (BC-3) (Shinya et al., 2014) precultivated on MEA (Difco™ Malt Extract Agar) at 20°C.

### Validation of RNA-Seq by QRT-PCR

To validate RNA-seq-based induction of CLT1 and CLT2, quantitative real-time PCR (qRT-PCR) was performed with three biological replicates of RNA. A small chunk of mycelium from *C. cinerea* AmutBmut was inoculated into microfluidic devices and cultivated for 30 h at 37°C. Subsequently, roughly 10 fungivorous nematode *Aphelenchus avenae* were added to the interaction zones and the microfluidic devices were incubated for another 8 h at 20°C. Thereafter, mycelia was extracted from the interaction zone for RNA extraction. RNA was extracted using Norgen RNA extraction kit according to the manufacturer's protocol (Norgen Biotek Corporation, Canada). For each sample, 15 ng of extracted RNA was reverse transcribed into cDNA using the Transcriptor Universal cDNA Master (Roche, Switzerland) following the manufacturer's instructions. Twenty microlitre qRT-PCR reactions containing 2 μl of cDNA, 10 μl 2x FastStart Universal SYBR Green Master (Roche, Switzerland) and 900 nM of the respective primer pair were prepared. qRT-PCR was performed in a Rotor-Gene 3000 (Corbett Life Science, Australia) in quadruplicates for each biological replicate with following program: 95°C for 15 min followed by denaturation at 95°C for 15 s, annealing at 60°C for 30 s and extension at 72°C for 30 s (40 cycles). The specificity of amplification was confirmed with melting curve analysis. Differential gene expression ratios were calculated using the CT formula (Schefe et al., 2006). Primers were designed at the Primer3Plus website (Untergasser et al., 2007) where at least one primer of each pair was designed to span exon-exon junctions. Samples that were not challenged with nematode were used as controls. The housekeeping gene tubulin (MycoCosm JGI protein ID: 357668) was used as an internal standard. qRT-PCR primers are listed in Supplementary Table 2.

### Construction of Expression Plasmids Harboring CLT1- and CLT2- Encoding cDNAs

The cDNA synthesized from RNA of nematode-induced *C. cinerea* was used as a template for the amplification of the coding region of CLT1 and CLT2 by PCR. The primer pairs used for cloning of the coding regions are given in Supplementary Table 2. The obtained PCR products were cloned into *E. coli* expression vector pET-24b (+) (Novagen, Germany) using *Nde*I/*Vsp*I and *Not*I restriction sites. The constructs were verified by Sanger sequencing (Microsynth, Switzerland), and transformed into *E. coli* BL21 for heterologous expression. Transformants were cultured in LB medium supplemented with 50 mg/l kanamycin at 37°C. Cultures were induced with 0.5 mM isopropyl β-D-1-thiogalactopyranoside (IPTG) at OD_600_ = 0.5 and cultivated overnight at 18°C. Heterologous expression and solubility for CLT1 and CLT2 were assessed as previously described (Kunzler et al., 2010).

### Toxicity of CLT1 and CLT2 Toward Nematodes and Insects

To assess nemato- and entomotoxicity of the heterologously expressed lipases, seven different species of bacterivorous nematodes and the omnivorous mosquito *Aedes aegypti* (see Supplementary Table 1), respectively, were used. For nematotoxicity assays, nematode eggs were isolated and hatched to L1 larvae as described in the wormbook (Stiernagle, 2006). Twenty to thirty freshly hatched, synchronized L1 stage-larvae were added to 100 μl of PBS containing preinduced *E. coli* BL21 cells adjusted to OD_600_ 2.0. After 48 h (72 h for *H. gingivalis*) of incubation at 20°C, the percentage of nematodes that developed into L4 larvae or adulthood were assessed. Toxicity of CLT1 and CLT2 toward *Aedes aegypti* larvae was assayed as previously described (Kunzler et al., 2010). *E. coli* BL21 cells expressing previously characterized, nemato- and entomotoxic lectin CGL2 was used as a positive control in all toxicity assays, and BL21 cells carrying vector without insert (empty vector) served as a negative control. All assays were performed in three or four biological replicates. Dunnett's multiple comparisons test was used for statistical analysis.

### Tagging and Purification of the Proteins

To test the *in vitro* lipase activity of CLT1 and CLT2, the proteins were tagged at their N-termini with 8 His-residues and purified on Ni-NTA columns as described previously (Bleuler-Martinez et al., 2011). In brief, the parental expression plasmids encoding untagged CLT1 and CLT2 were PCR-amplified using 8-histidine tag-encoding primers (Supplementary Table 2). After the PCR, the reaction products were treated with methylation-dependent *Dpn*I endonuclease to eliminate the plasmid template. Five microlitre of the treated PCR product was ligated and transformed into *E. coli* DH5α cells. The retrieved plasmids were verified with Sanger sequencing and transformed into *E. coli* BL21 for protein expression and purification. The proteins were expressed as described above for the untagged lipase proteins. The protein-expressing bacterial cells were centrifuged and resuspended in lysis buffer (50 mM Tris-HCI, 5 mM imidazole, pH 8.5) before being lysed using a French press. The bacterial lysate was spun at 16,000 rpm for 30 min at 4°C and the supernatant containing the soluble fraction was incubated with Ni-NTA beads (Macherey-Nagel, Germany) overnight at 4°C. The beads were washed with lysis buffer and subsequently eluted with elution buffer (50 mM Tris-HCI, 250 mM imidazole, pH 8.5). The final eluate was desalted and concentrated on a disposable PD-10 Desalting column (GE Healthcare Life Sciences™, USA).

### Construction and Expression of Catalytic Site Mutants and Truncated Constructs

Mutations in the putative catalytic sites of CLT1 and CLT2 were introduced by PCR. The expression plasmids encoding His8-tagged CLT1 and CLT2 were amplified with mutagenic PCR primers carrying the respective mutations (Supplementary Table 2). Likewise, the N- and C- termini of CLT1 and CLT2 were truncated by PCR using the specific primers listed in Supplementary Table 2. Cloning of the plasmids and protein expression and purification were performed as described above.

### *In vitro* Lipase Assays

*In vitro* lipase activities of CLT1 and CLT2 were assayed by measuring the rate of release of *p-*nitrophenol from *p*-nitrophenyl acetate (C2), *p*-nitrophenyl butyrate (C4), and *p*-nitrophenyl palmitate (C16) (all from Sigma) (Glogauer et al., 2011). Two microgram of purified protein was mixed with each substrate (20 mM) in 200 μl reaction buffer [100 mM phosphate buffer, 150 mM NaCl and 0.5% (v/v) Triton X−100, pH 7.2]. The reaction was monitored in the microplate reader (Infinite 200 PRO; Tecan) at 25°C by measuring the OD405 in time intervals of 3–150 min, depending on the activity of the lipases for the individual substrates. *Candida rugosa* lipase (Sigma) was used as a positive control to evaluate triacylglycerols as substrates.

## Results

### Identification of Candidate Nematotoxic Proteins From *C. cinerea* Based on Differential Gene Expression

**Figure 1:**
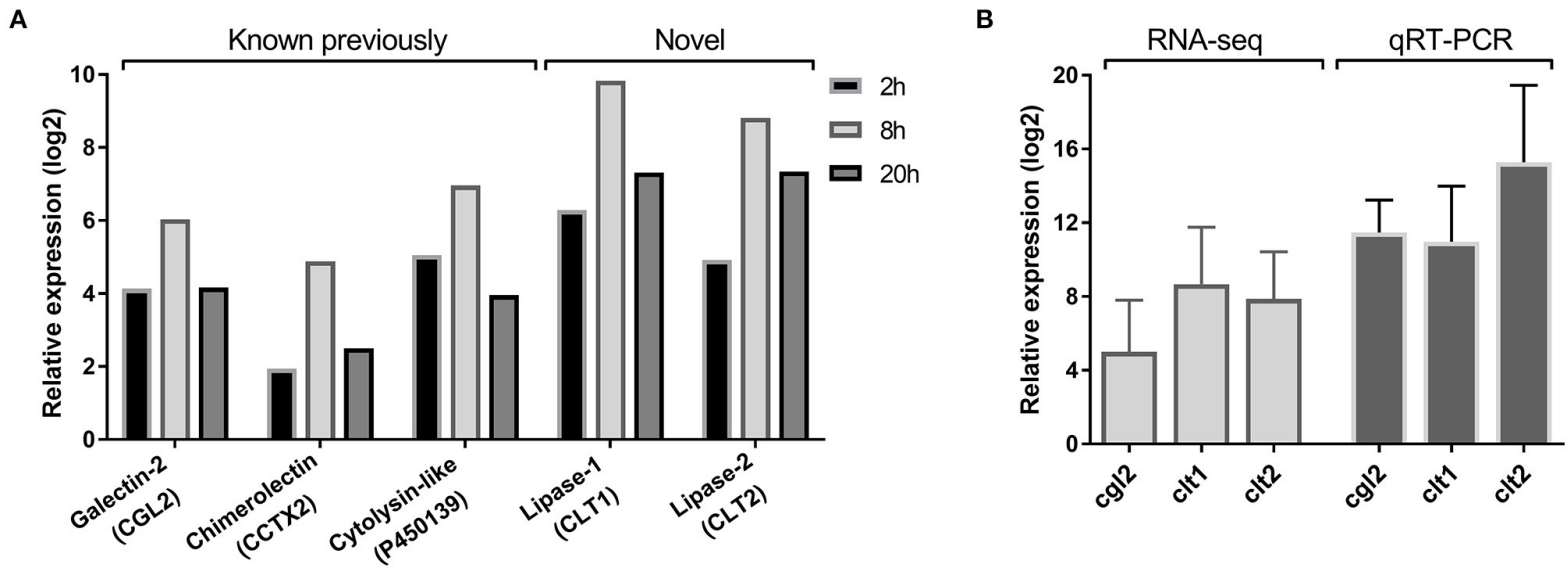
Nematode-inducible defense proteins of *C. cinerea*. **(A)** RNA-seq based expression pattern of previously known and novel defense proteins of *C. cinerea* challenged by *A. avenae* for indicated time periods. Excluding CCTX2 at 2 h, all genes were significantly differentially upregulated (log2 Fold Change >2, False Discovery Rate <0.05) in nematode-treated samples compared to the control samples. **(B)** Expression of novel defense proteins along with CGL2 as a positive control validated with qRT-PCR. *C. cinerea* was challenged by *A. avenae* in a microfluidic device for 8 h. Error bars represent the standard deviation of three biological replicates.

Previous sequencing of the *C. cinerea* transcriptome upon challenge with the fungivorous nematode *A. avenae* resulted in the identification of over one thousand nematode-induced *C. cinerea* genes (Tayyrov et al., 2019b). Two of these genes, *clt1*, and *clt2*, showed a similar expression pattern regarding the different cocultivation periods as some previously characterized nematotoxic proteins (Figure 1A). To confirm the induction of these two genes upon nematode challenge, we performed qRT-PCR using RNA of *A. avenae*-induced mycelia of *C. cinerea*. Both *clt1* and *clt2* revealed strong upregulation in nematode-challenged samples compared to non-challenged samples, confirming the RNA-seq results (Figure 1B).

### CLT1 and CLT2 Contain Lipase Domains

**Figure 2:**
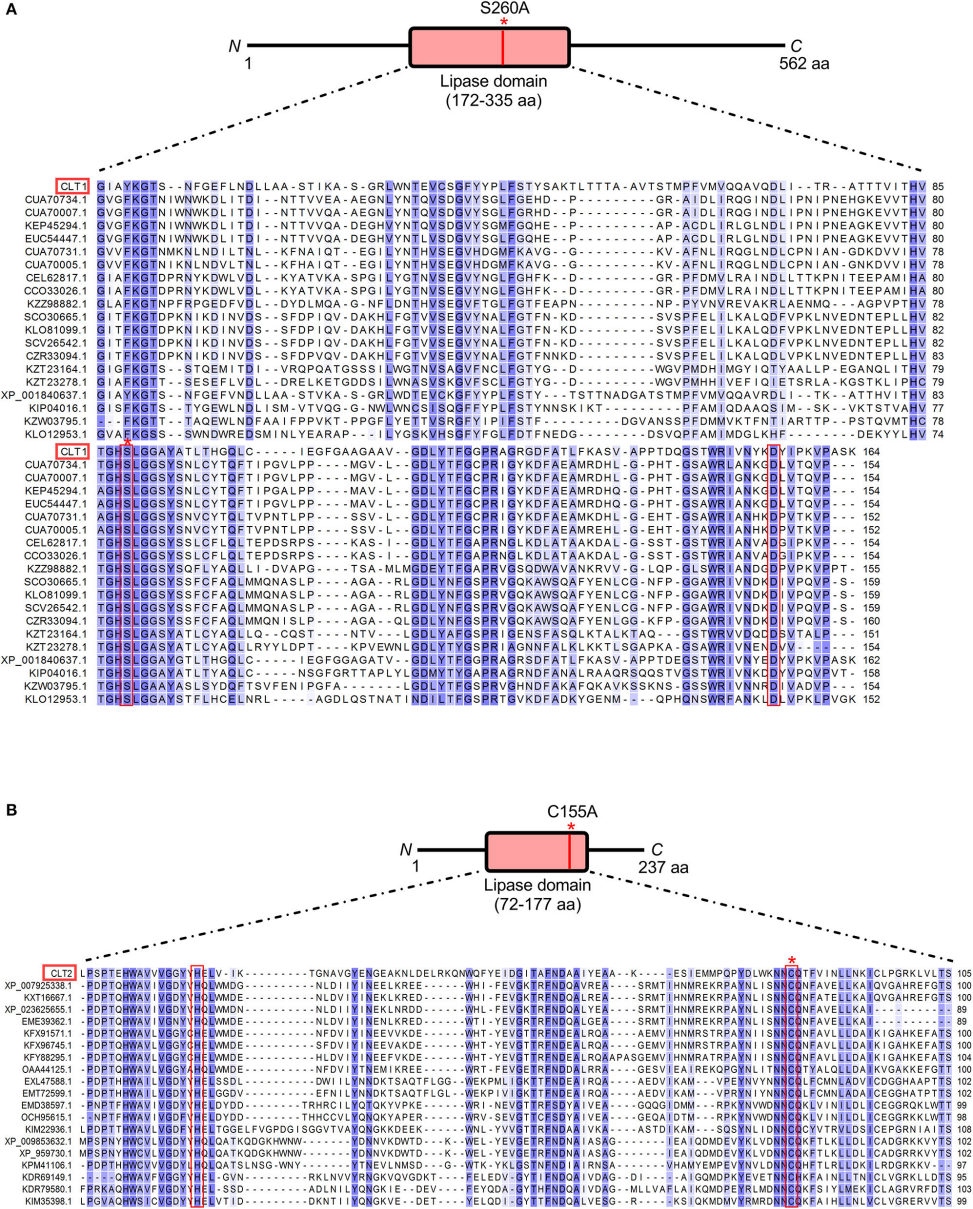
Sequence alignments of CLT1 and CLT2 lipase domains with homologous sequences from NCBI. Amino acid sequences of CLT1 **(A)** and CLT2 **(B)** lipase domains were BLASTed against the NCBI non-redundant protein sequence database and the top 20 hits were aligned. All hits originate from fungi and 33 of them are annotated as an uncharacterized protein, three as a lipase and four as a hydrolase. Blue shading indicates the degree of conservation from dark (highly) to light (low). The designations of the two potential lipases and their putative catalytic dyads are denoted by red line boxes. Mutated putative catalytic residues, S266 for CLT1 and C155 for CLT2, are indicated with an asterisk. N—and C—termini are labeled. The length of the entire proteins and the lipase domains are indicated with amino acid location numbers. The sequences were aligned using the ClustalW algorithm (v2.1). The originating species, the annotations and the amino acid sequences of the aligned proteins can be found in Supplementary File 2.

The predicted amino acid sequences of CLT1 and CLT2 (Supplementary File 2) were examined by SMART (Simple Modular Architecture Research Tool) (Letunic and Bork, 2018) and *Phyre2* (Kelley et al., 2015) web servers for characterized protein domains based on sequence and structural homologies, respectively. The search results indicated that both proteins are localized in the cytoplasm based on the absence of any secretion or subcellular localization signals. CLT1 contains a putative lipase domain covering the region from residues 172–335 including conserved serine-aspartate residues forming a putative catalytic dyad for serine-dependent hydrolysis of ester bonds between fatty acids and glycerol (Sato et al., 2003; Simon and Cravatt, 2010; Agarwal et al., 2015) (Figure 2A). Similarly, the CLT2 homology search predicted a putative (phospho)lipase domain in the region between residues 72 and 177. This predicted lipase domain contains conserved histidine-cysteine residues known to form the catalytic site for cysteine-dependent hydrolysis of (phospho)glycerolipids (Uyama et al., 2009) (Figure 2B). Besides containing a predicted lipase domain, the amino acid sequences of CLT1 and CLT2 do not share any significant similarity.

### Heterologous Expression and Nematotoxicity of CLT1 and CLT2

**Figure 3:**
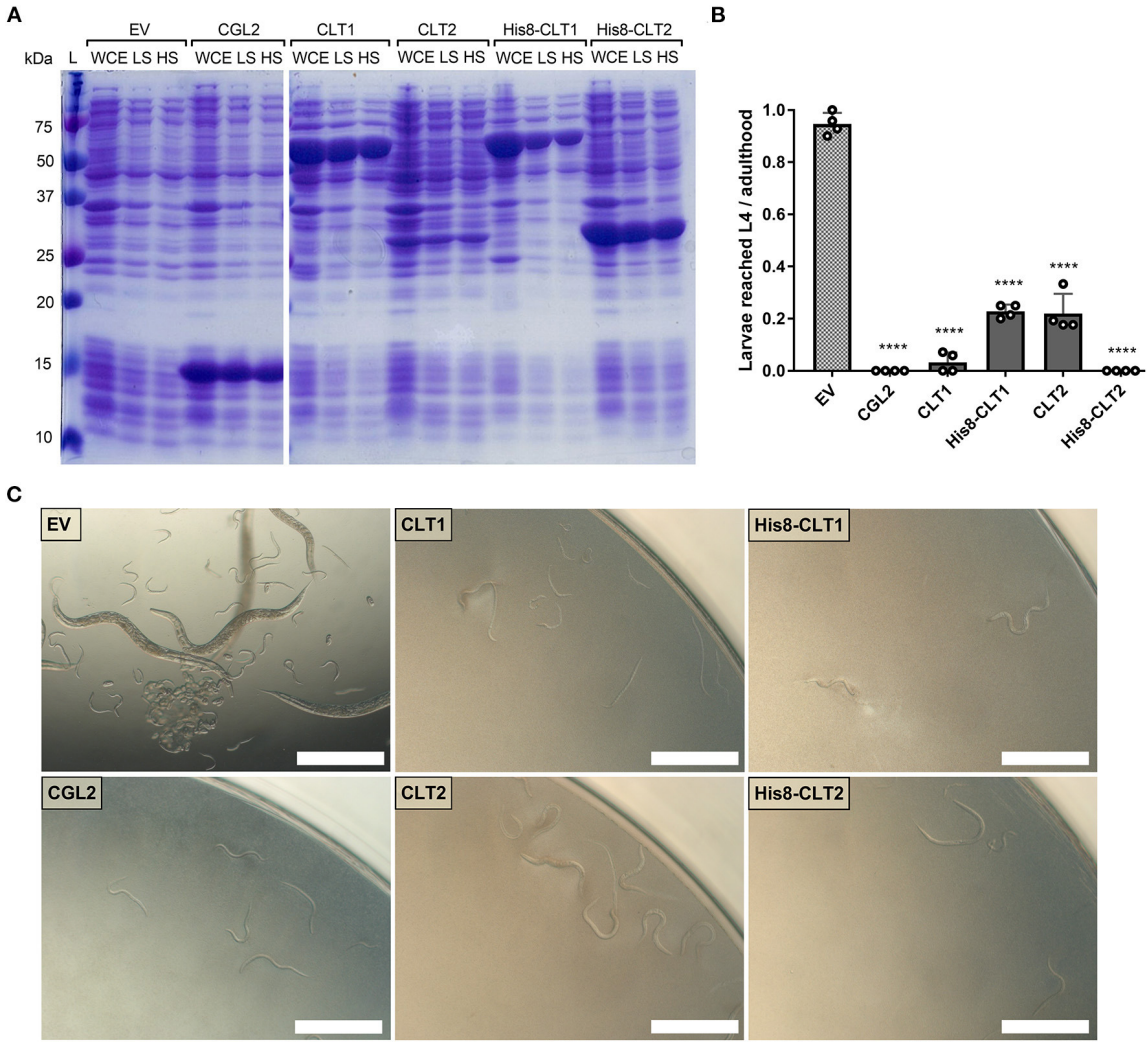
CLT1 and CLT2 are nematotoxic proteins with predicted lipase domains. **(A)** Coomassie-stained SDS-PAGE showing heterologous expression and solubility of wild-type and His8-tagged CLT1 and CLT2 proteins. Twenty microliters of whole-cell extract (WCE) and supernatants of low-spin (LS; 5min. at 5,000 g) and high-spin (HS; 30 min. at 16,000 g) bacterial lysate were loaded on a gel. CGL2 was used as a positive control and an “empty” vector (EV) was used as a background control for IPTG-induced expression and solubility. **(B)** Toxicity of untagged CLT1 and CLT2 as well as of their His8-tagged versions against *C. elegans* N2. IPTG-induced *E. coli* BL21 expressing previously characterized nematotoxic protein CGL2 and containing an “empty” vector (EV) were used as positive and negative controls, respectively. Dunnett's multiple comparisons test was used for statistical analysis. Error bars represent the standard deviation of four biological replicates. ^*^p < 0.05, **p < 0.01, ***p < 0.001, ****p < 0.0001 vs. EV. **(C)** Phase-contrast micrographs of *C. elegans* fed with IPTG-induced *E. coli* BL21 for 72 h expressing either of the indicated constructs. Scale bar = 500 μm.

In order to functionally characterize CLT1 and CLT2, the untagged and His8-tagged versions of the proteins were expressed in *Escherichia coli*. Expression and solubility assays revealed high expression of the proteins in a largely soluble form (Figure 3A). We tested a possible nematotoxicity of these proteins by feeding *Caenorhabditis elegans* L1-staged larvae with *E. coli* BL21 cells expressing the proteins of interest. After 48 h of incubation, L4 larvae and adult nematodes were counted. The results showed strong toxicity of both CLT1 and CLT2 against the model bacterivorous nematode (Figures 3B,C). Higher expression levels of His8-tagged vs. untagged CLT2 (Figure 3A) correlated with higher nematotoxicity (Figure 3B). In the case of CLT1, the tagging decreased the solubility of the protein (Figure 3A), and, accordingly, the toxicity of His8-tagged CLT1 was less severe than the untagged form (Figure 3B). The correlation between the amount of the expressed protein and the degree of toxicity suggests that toxicity is dependent on the concentration of soluble protein as previously suggested for other protein toxins (Kunzler et al., 2010).

### *In vitro* Lipase Activity of CLT1 and CLT2 and Mutational Analysis of the Putative Catalytic Residues

**Figure 4:**
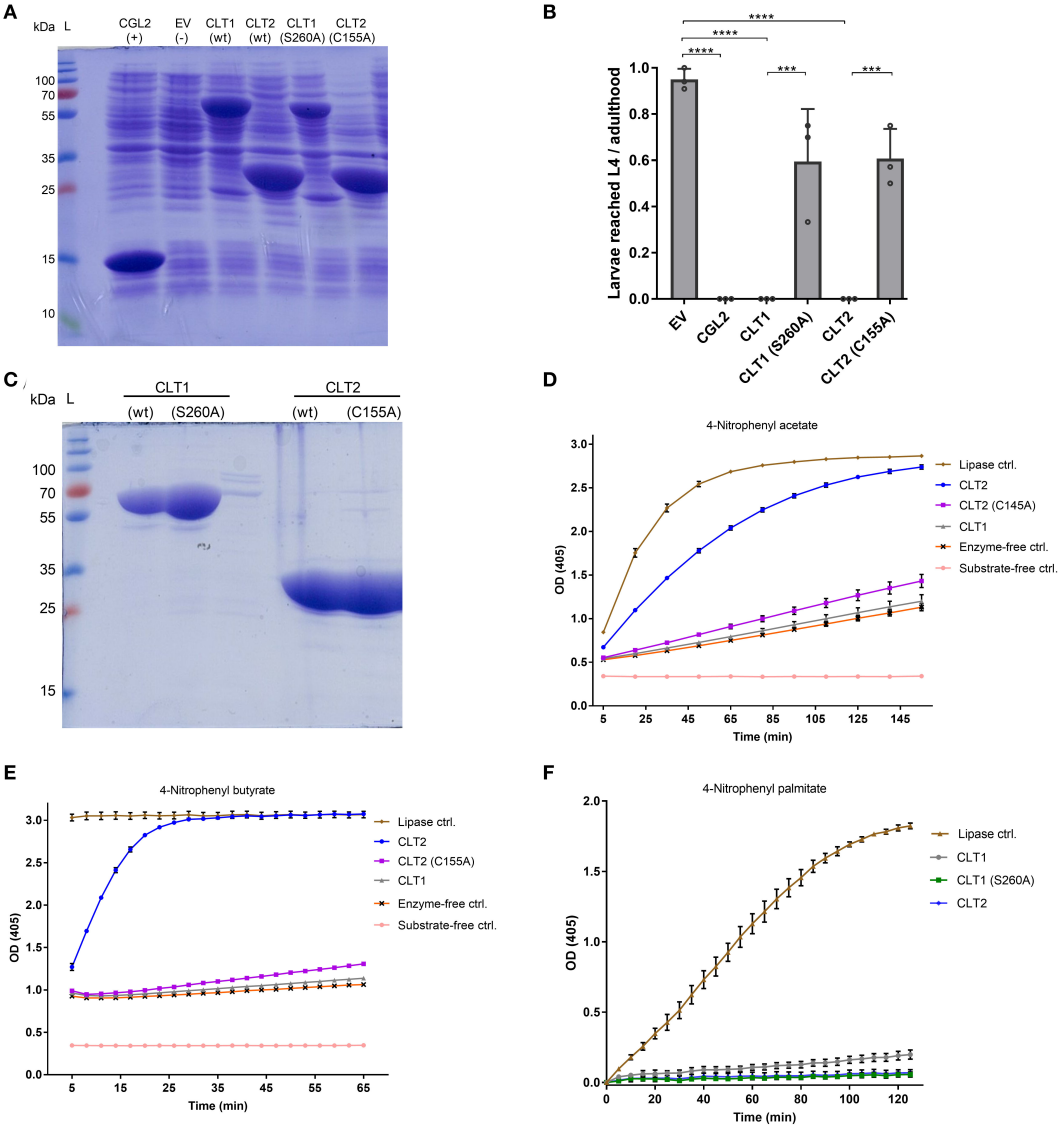
*In vitro* lipase activity of wild type CLT1 and CLT2 proteins and their putative catalytic site mutants. **(A)** Heterologous expression of N-terminally Hi8-tagged, wild type CLT1 and CLT2 proteins and their putative catalytic site mutants. Twenty microliter of bacterial lysate was loaded on a SDS-PAGE and stained with Coomassie blue. CGL2 and “empty” vector (EV) was used as positive and background controls for IPTG-induced expression, respectively. **(B)** Effect of catalytic site mutations on the toxicity of CLT1 and CLT2 against *C. elegans* N2. IPTG-induced *E. coli* BL21 expressing previously characterized nematotoxic protein CGL2 and containing “empty” vector (EV) were used as positive and negative controls, respectively. Dunnett's multiple comparisons test was used for statistical analysis. Error bars represent the standard deviation of three biological replicates. ****p < 0.0001 vs. EV. **(C)** The recombinant proteins were purified over Ni-NTA beads and 10 μl of the final eluate were run out on a SDS-PAGE followed by Coomassie staining. The masses of the molecular marker proteins are indicated. Lipase activity of the purified proteins was assessed toward *p*-nitrophenyl acetate **(D)**, *p*-nitrophenyl butyrate **(E)**, and *p*-nitrophenyl palmitate **(F)**. Enzyme activity is shown as mean values ± SD (*n* = 4) for each time point. The error bars indicate standard deviation of four technical replicates. The error bars were not drawn if they are shorter than the height of the symbol. *Candida rugosa* lipase (L1754, Sigma) was used as a positive control and labeled as “Lipase ctrl”.

In order to confirm the predicted lipase activity of CLT1 and CLT2, we expressed and purified His8-tagged derivatives of wild-type CLT1 and CLT2 from *E. coli* (Figure 4C). The purified recombinant proteins were incubated with three different chromogenic substrates, and the lipase (esterase) activity of the proteins was determined by the release of *p*-nitrophenol. In these assays, CLT2 showed lipase activity with *p*-nitrophenyl acetate (Figure 4D) and p-nitrophenyl butyrate (Figure 4E). Interestingly at the same concentration, we did not detect CLT2 activity against a longer carbon chain ester *p*-nitrophenyl palmitate (Figure 4F). However, when the concentration of CLT2 was increased from 10 to 800 ng/ul weak activity was observed against *p*-nitrophenyl palmitate (Supplementary Figure 1). Conversely, CLT1 was neither active toward *p*-nitrophenyl acetate (Figure 4D) nor toward *p*-nitrophenyl butyrate (Figure 4E). The protein showed, however, weak activity toward *p*-nitrophenyl palmitate (Figure 4F).

As additional confirmation of these lipase activities, we introduced mutations in the putative catalytic site residues of the toxins. Amino acid sequences of the predicted lipase domains of CLT1 and CLT2 were BLASTed against the NCBI non-redundant protein sequence database. Interestingly, exclusively domains of fungal proteins, mostly annotated as hypothetical proteins, including a non-induced paralog of CLT1 (NCBI XP_001840637.1), were identified. Alignment of the top 100 sequences revealed presumed serine-aspartate catalytic dyads (Simon and Cravatt, 2010) for CLT1. Both S260 and D326 amino acid residues were found to be conserved within 99 of the top 100 sequences. A similar analysis for CLT2 showed that putative catalytic dyads H89 and C155 (Uyama et al., 2009) were conserved for 99 and 100 of the top 100 hits, respectively. The alignments of the top 20 sequences are shown in Figures 2A,B.

In order to assess the function of the identified putative catalytic residues, CLT1(S260) and CLT2(C155) of the His8-tagged proteins were mutated to alanine. Subsequent SDS-PAGE analysis demonstrated that both toxin variants were expressed in good amounts and soluble form in *E. coli* (Figure 4A). *In vitro* lipase assays with the toxin variants revealed that the lipase activity of CLT2(C155A) was almost completely abolished against both substrates. The weak activity of CLT1 toward *p*-nitrophenyl palmitate was abolished by mutation of the predicted catalytic residue (S260A) demonstrating that this activity is significant.

Nematotoxicity assays with *C. elegans* showed that the mutations of the predicted catalytic residues significantly reduced the toxicity for both CLT1 and CLT2 (Figure 4B). Taken together, these data suggest that CLT1 and CLT2 are lipases and that their catalytic activity is required for their nematotoxicity.

### Toxicity Spectrum of CLT1 and CLT2

**Figure 5:**
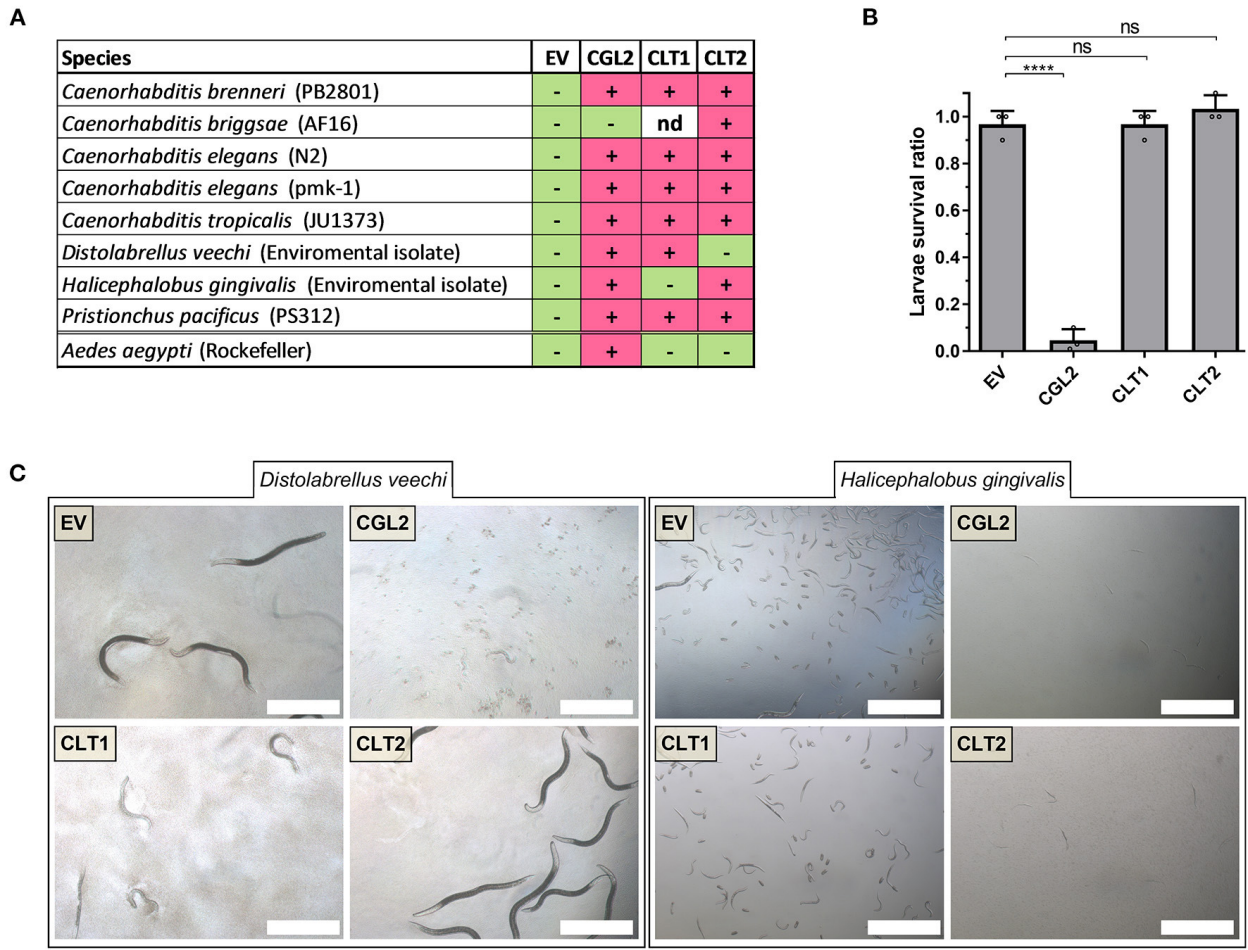
Toxicity of CLT1 and CLT2 against bacterivorous nematodes and an omnivorous insect. **(A)** Toxicity spectrum of CLT1 and CLT2 proteins was assessed against seven different species of bacterivorous nematodes and larvae of the mosquito *Aedes aegypti*. Minus (–), lack of toxicity; plus (+), presence of toxicity; nd, not determined. **(B)** Toxicity of CLT1 and CLT2 against *A. aegypti* larvae was quantified by counting number of survived larvae after 4 days of feeding on IPTG-induced *E. coli* BL21 bearing CLT1 or CLT2. *E. coli* BL21 expressing either previously characterized nematotoxic protein CGL2 or carrying an “empty” vector (EV) were used as positive and negative controls, respectively. Dunnett's multiple comparisons test was used for statistical analysis. Error bars represent standard deviation of three biological replicates. ***p < 0.001, ****p < 0.0001 vs. EV. **(C)** Differential susceptibility of *D. veechi* and *H. gingivalis* against two lipases when they were fed with IPTG-induced *E. coli* BL21 for 72 h containing either an “empty” vector (EV) or expressing CGL2 or CLT1 or CLT2. Scale bar = 500 μm.

After confirming the toxicity of CLT1 and CLT2 toward the model organism *C. elegans*, we tested their toxicity against six other different bacterivorous nematode species and one omnivorous insect in order to assess the activity spectrum of these lipase toxins. The results are summarized in Figure 5A. In contrast to the previously characterized lectin CGL2, neither CLT1 nor CLT2 showed toxicity against larvae of the mosquito *Aedes aegypti* (Figure 5B). Regarding the tested bacterivorous nematodes, CLT1 and CLT2 showed differences in their activity spectrum; while CLT1 was toxic toward *Distolabrellus veechi*, CLT2 was ineffective toward the same nematode species. The toxicity of CLT1 against *D. veechi* was weaker than the toxicity of CGL2 (Figure 5C). We also found a different susceptibility of the facultative parasitic nematode *Halicephalobus gingivalis* toward the two lipase toxins. In this case, CLT2 was toxic whereas no toxicity of CLT1 toward the same nematode was detected (Figure 5C). These results suggest differences in the accessibility, or the levels of the lipids targeted by these toxins between the different nematode species.

## Discussion

In this study, we report the characterization of two novel nematotoxic proteins, CLT1 and CLT2, containing predicted lipase domains from the multicellular fungus *C. cinerea*. The genes coding for these fungal proteins were transcriptionally upregulated upon nematode predation, which indicates the involvement of these proteins in the inducible defense of the fungus against nematodes (Caballero Ortiz et al., 2013; Mathioni et al., 2013; Plaza et al., 2015). Interestingly, a paralog of CLT1 (NCBI XP_001840637.1) is not induced by nematodes but during fruiting body development (Plaza et al., 2014). This expression pattern results in an inducible protection of the vegetative mycelium and a constitutive protection of the fruiting body from predation by nematodes. Similar expression patterns have been observed for other nematotoxic defense proteins from *C. cinerea* (Tayyrov et al., 2019b).

The results of our biochemical and mutational studies indicate that the nematotoxicity of the two identified *C. cinerea* proteins, CLT1 and CLT2, is dependent on their lipase (esterase) activity, and that residues S260 and C155 are part of the respective catalytic (active) sites. Mutation of analogous residues has shown to be essential for the function of other lipases; Sato et al. showed that mutating the predicted active site residue (S142) of the *P. aeruginosa* lipase toxin ExoU inhibits its cytotoxicity and reduces the release of palmitic acid through *in vivo* assays (Sato et al., 2003; Sato and Frank, 2004). Another study showed that mutating the cysteine residue in the catalytic dyad of the cytotoxic tumor suppressor protein H-Rev107 eliminates its phospholipase activity along with its cytotoxicity (Uyama et al., 2009; Wei et al., 2015). Additional biochemical studies will be needed to determine the substrate specificities of the two lipases.

In addition to the *C. elegans* toxicity assay, we tested CLT1 and CLT2 for toxicity against seven other nematode species, including mosquito larvae, in order to determine the activity spectrum of these toxins. In contrast to the positive control, the lectin CGL2, neither CLT1 nor CLT2 was active against *A. aegypti* larvae; this could mean that this insect does not contain any target molecules for these toxins or that they were not accessible to the toxins. Interestingly, we also observed a difference in the nematotoxicity of CLT1 and CLT2 against two of the seven tested species. Even though both are lipase domain-containing toxins, only CLT1 was toxic against the free-living bacterivorous nematode *D. veechi* and only CLT2 was toxic against the parasitic nematode *H. gingivalis*. These findings suggest that these toxins have different targets. Several studies have shown different lipid compositions within different nematode species and proposed the identification of organisms based on their unique lipid profiles (Sekora et al., 2009; Kühn et al., 2018). These differences in lipid compositions and the substrate specificities of CLT1 and CLT2, may explain the specific toxicity of the fungal lipase toxins toward certain species.

One of the main questions that remains unanswered in our study is the self-protection mechanism of the producer fungus against its cytoplasmic lipase toxins. There are three main ways by which producers can overcome self-intoxication by their toxins; (a) they co-express a specific antitoxin along with a toxin (Munoz-Gomez et al., 2005; Campos et al., 2016), (b) they express the toxin in inactive form that requires host-specific cofactors to activate the toxin (Christen et al., 2009; Tyson and Hauser, 2013; Anderson et al., 2015), (c) the target molecule of the toxin is missing or inaccessible in the producer (Butschi et al., 2010). Several studies have failed in demonstrating *in vitro* activity of purified recombinant lipase toxins indicative of a need for activation by the host (Anderson et al., 2011). However, in our study, we were able to detect lipase activity against synthetic lipid substrates (esters), especially in the case of CLT2, which makes this resistance mechanism appear unlikely. Lipidomic studies have, however, revealed the unique composition of fatty acids for different species of fungivorous nematodes and fungi (Chen et al., 2001). These results suggest that the fungus protects itself from its lipase toxins by adjusting the specificity of the toxins toward the lipids of the antagonist. In agreement with this hypothesis, the CLT1 and CLT2 proteins can be expressed in the cytoplasm of *E. coli* in soluble form without any sign of toxicity against the expressing bacteria. These results indicate that these lipase toxins have a pronounced substrate specificity and do not affect the inner leaflet of the *E. coli* or *C. cinerea* plasma membrane. The observed differences in the activities of CLT1 and CLT2 against different substrates in our *in vitro* lipase assays support the pronounced substrate specificity of these lipase toxins.

Proteins with homology to CLT1 and CLT2 were only found in fungi and outside of the predicted lipase domains, CLT1 and CLT2 lack significant sequence homology with known lipases. These results indicate that these toxins may represent previously uncharacterized, fungal-specific lipase families. As seen in Figure 2, the predicted lipase domain is roughly one-third of the whole protein sequence of both CLT1 and CLT2. Therefore, we aimed at testing the importance of the regions outside of this predicted lipase domain for the nematotoxicity and the *in vitro* lipase activity of CLT1 and CLT2. In the case of other lipase toxins, the N- and C-terminal regions are postulated to be involved in activation of the lipase activity of the toxin upon binding of a host factor, proteolytic cleavage, or subcellular localization of the toxin (Sato et al., 2003; Wei et al., 2015). Therefore, we truncated CLT1 and CLT2 N- and C-terminally and cloned them into *E. coli* BL21 for expression and subsequent activity studies. Unfortunately, these constructs (CLT1ΔC, CLT1ΔN, CLT2ΔC, and CLT2ΔN) were either insoluble or toxic to *E. coli* upon induction of their expression (Supplementary Figure 2). Therefore, we could not proceed with the activity studies, and the roles of the N- and C- terminal regions of CLT1 and CLT2 remain to be elucidated. The toxicity of the expression of some of these proteins may suggest that the toxicity of these lipase toxins might not solely be controlled by their substrate specificity.

Nematodes are responsible for several human and animal diseases (Waller, 2003; L'ollivier and Piarroux, 2013) and are one of the prominent pests in agriculture (Quist et al., 2015; Engelbrecht et al., 2018). There are only few agents that can be used for the bio-control of nematodes (Witty, 1999; Kenney and Eleftherianos, 2016). Hence, our current findings may be useful for the development of potential control strategies for nematode-borne diseases of humans, animals, and crop plants.

## Data Availability Statement

The datasets referred to in this study can be found in the online repository ArrayExpress (https://www.ebi.ac.uk/arrayexpress/) under accession number E-MTAB-7005.

## Author Contributions

AT and MK: conceptualization. AT, CW, CF, AG, and PS: methodology, investigation, and data curation. AT: writing—original draft preparation. AT, MK, and LN: writing—review and editing. MK: supervision and funding acquisition. All authors have read and agreed to the published version of the manuscript.

## Conflict of Interest

The authors declare that the research was conducted in the absence of any commercial or financial relationships that could be construed as a potential conflict of interest.
