# Erratum: Cytoplasmic Lipases—A Novel Class of Fungal Defense Proteins Against Nematodes

**DOI:** 10.3389/ffunb.2022.910647

**Published:** 2022-04-28

**Authors:** Frontiers Production Office

**Affiliations:** Frontiers Media SA, Lausanne, Switzerland

**Keywords:** *Coprinopsis cinerea*, fungal toxins, toxic enzymes, inducible defense, nematotoxicity, esterase, active site residues

Due to a production error, in the original article, incorrect files were used for Figure 3 and Figure 5. The corrected Figure 3 and Figure 5 appear below.

**Figure 3:**
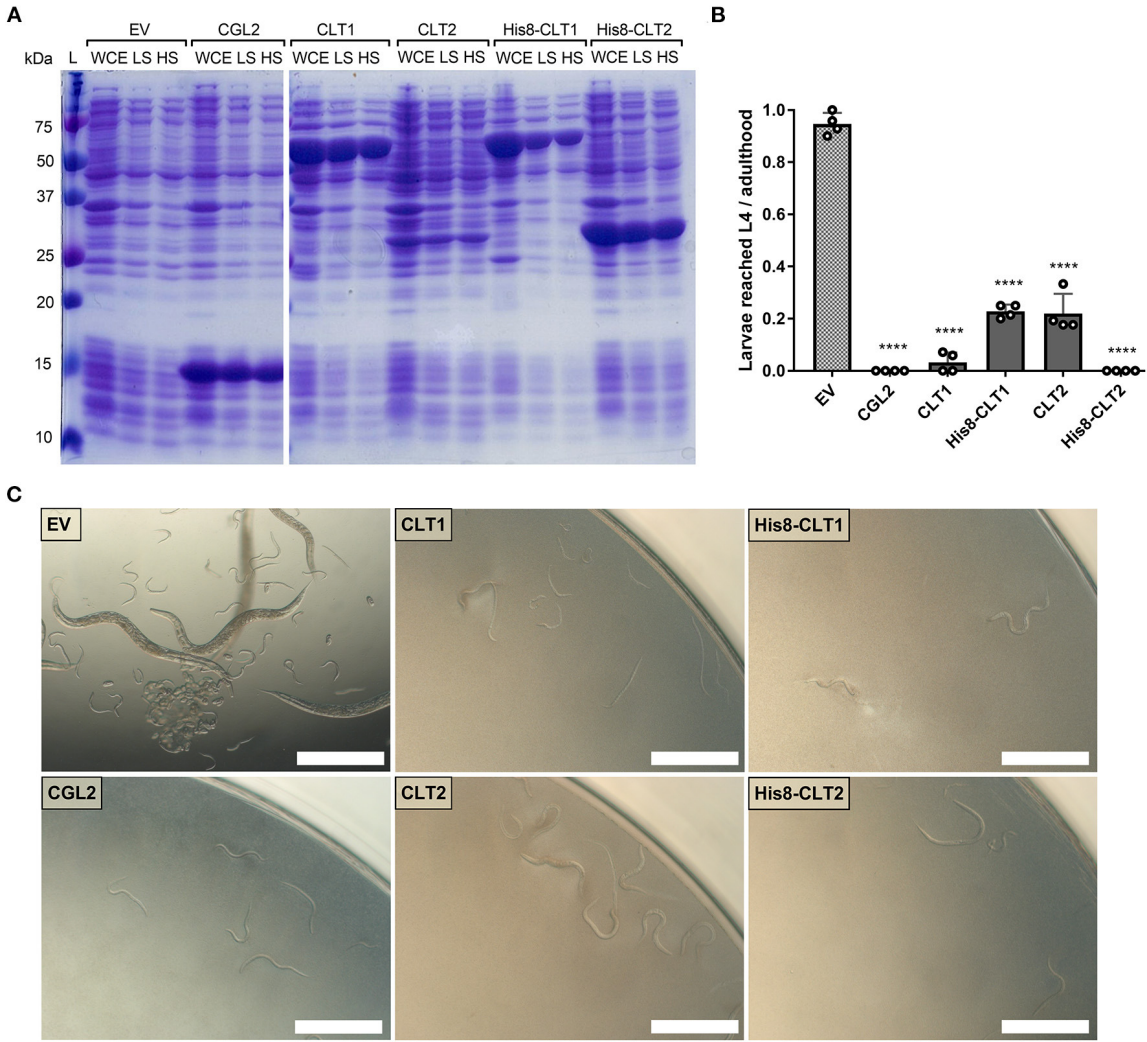
CLT1 and CLT2 are nematotoxic proteins with predicted lipase domains. **(A)** Coomassie-stained SDS-PAGE showing heterologous expression and solubility of wild-type and His8-tagged CLT1 and CLT2 proteins. Twenty microliters of whole-cell extract (WCE) and supernatants of low-spin (LS; 5min. at 5,000 g) and high-spin (HS; 30 min. at 16,000 g) bacterial lysate were loaded on a gel. CGL2 was used as a positive control and an “empty” vector (EV) was used as a background control for IPTG-induced expression and solubility. **(B)** Toxicity of untagged CLT1 and CLT2 as well as of their His8-tagged versions against *C. elegans* N2. IPTG-induced *E. coli* BL21 expressing previously characterized nematotoxic protein CGL2 and containing an “empty” vector (EV) were used as positive and negative controls, respectively. Dunnett's multiple comparisons test was used for statistical analysis. Error bars represent the standard deviation of four biological replicates. ^*^p < 0.05, **p < 0.01, ***p < 0.001, ****p < 0.0001 vs. EV. **(C)** Phase-contrast micrographs of *C. elegans* fed with IPTG-induced *E. coli* BL21 for 72 h expressing either of the indicated constructs. Scale bar = 500 μm.

**Figure 5:**
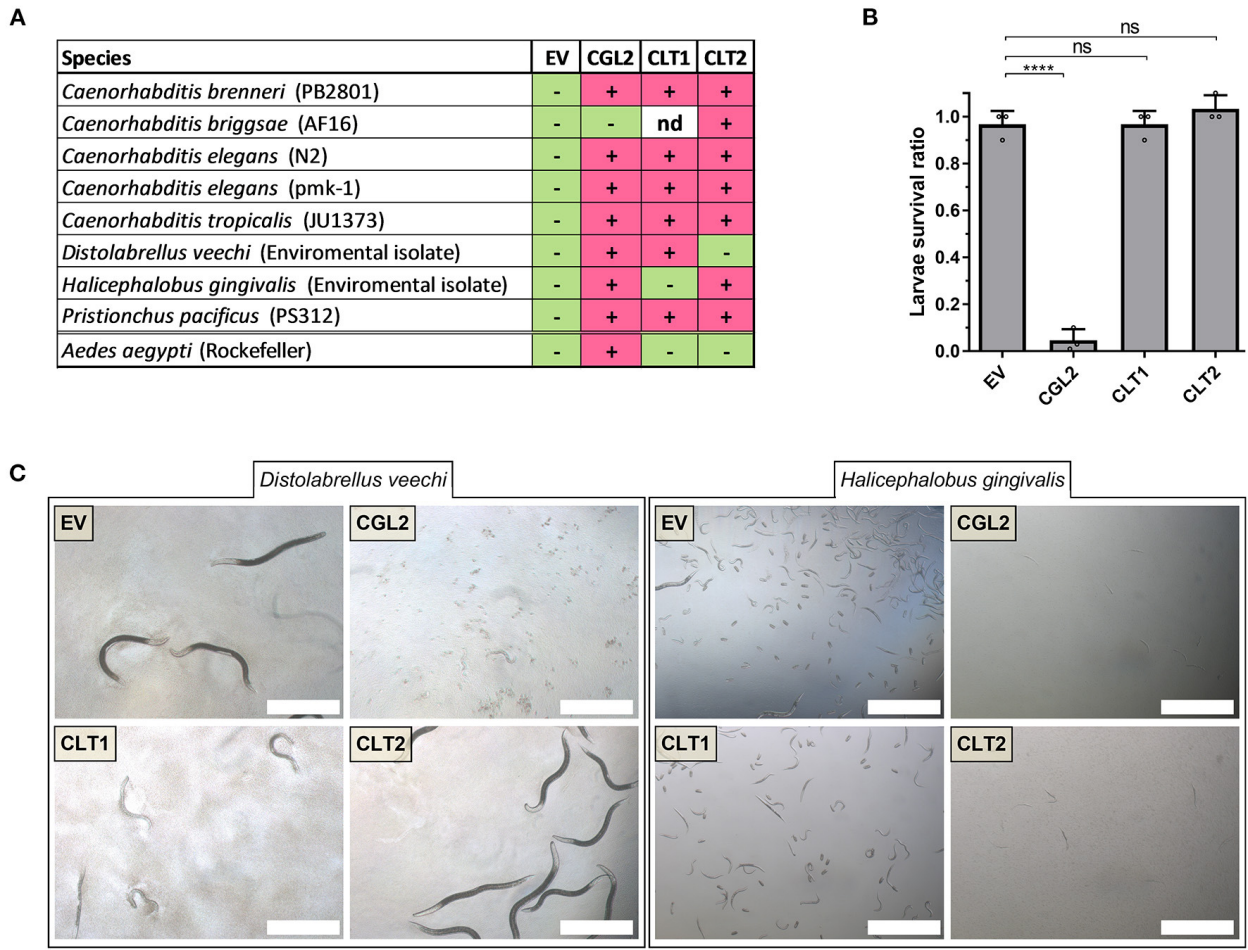
Toxicity of CLT1 and CLT2 against bacterivorous nematodes and an omnivorous insect. **(A)** Toxicity spectrum of CLT1 and CLT2 proteins was assessed against seven different species of bacterivorous nematodes and larvae of the mosquito *Aedes aegypti*. Minus (–), lack of toxicity; plus (+), presence of toxicity; nd, not determined. **(B)** Toxicity of CLT1 and CLT2 against *A. aegypti* larvae was quantified by counting number of survived larvae after 4 days of feeding on IPTG-induced *E. coli* BL21 bearing CLT1 or CLT2. *E. coli* BL21 expressing either previously characterized nematotoxic protein CGL2 or carrying an “empty” vector (EV) were used as positive and negative controls, respectively. Dunnett's multiple comparisons test was used for statistical analysis. Error bars represent standard deviation of three biological replicates. ***p < 0.001, ****p < 0.0001 vs. EV. **(C)** Differential susceptibility of *D. veechi* and *H. gingivalis* against two lipases when they were fed with IPTG-induced *E. coli* BL21 for 72 h containing either an “empty” vector (EV) or expressing CGL2 or CLT1 or CLT2. Scale bar = 500 μm.

The publisher apologizes for this mistake. The original article has been updated.

