# Supplementary material for: Cytoplasmic Lipases—A Novel Class of Fungal Defense Proteins Against Nematodes
Source: Front Fungal Biol. 2021 Jul 1;2:696972. doi: 10.3389/ffunb.2021.696972 (PMC10512399; doi:10.3389/ffunb.2021.696972)
Supplement: Supplementary file 2 [file Data_Sheet_1.PDF]

Supplementary Figures and Tables

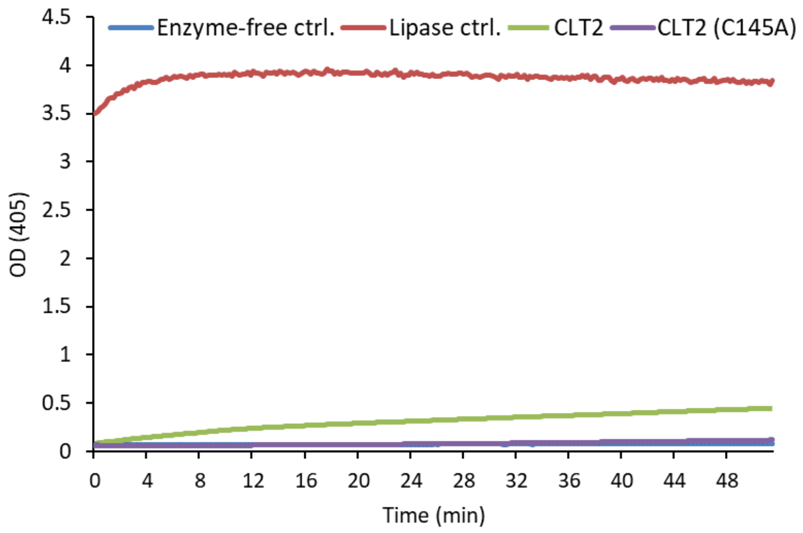

Figure S1: **Activity of CLT2 against *p*-nitrophenyl palmitate at high concentration.** Lipase activity of the purified CLT2 was assessed towards *p*-nitrophenyl palmitate using 800 ng/ul of the proteins. Enzyme activity is shown as mean values of three replicates for each time point. 'Lipase ctrl' is a positive control from *Candida rugosa* lipase (L1754, Sigma)

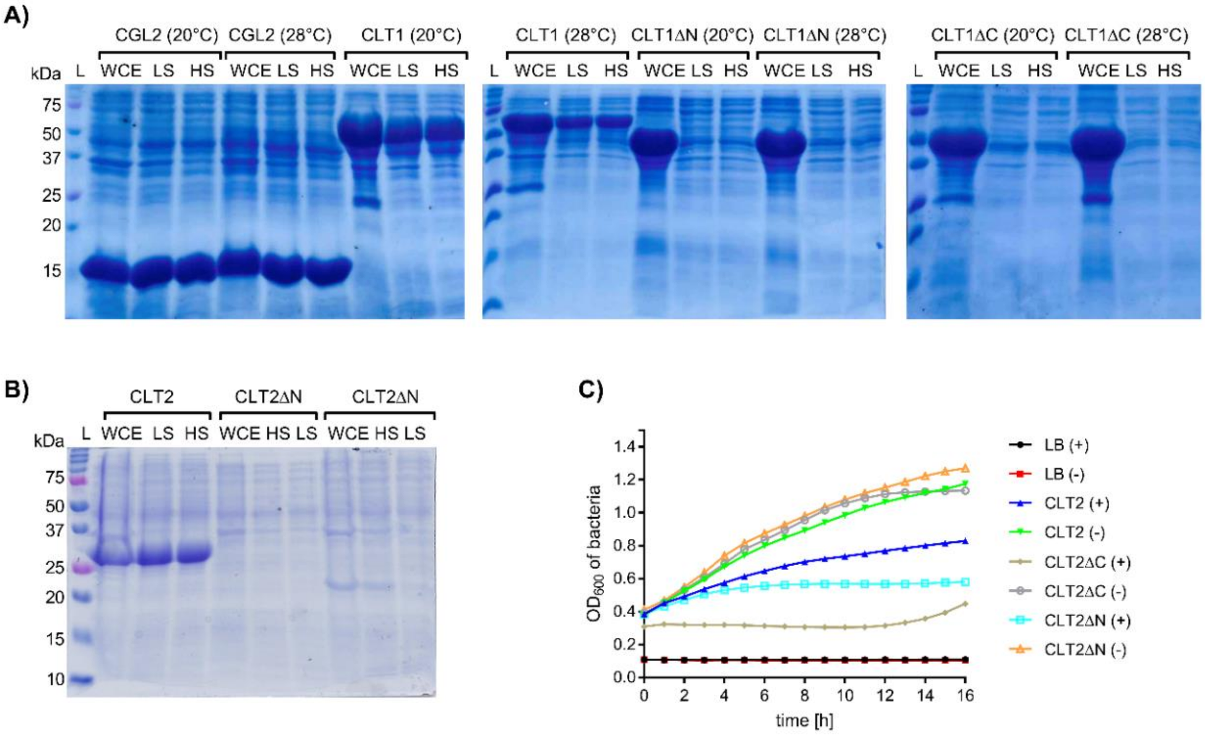

Figure S2: **Bacterial expression of truncated versions of CLT1 and CLT2. A)** Heterologous expression and solubility of CLT1 and its N- and C- termini truncated versions. 150 amino acids were truncated from either N-terminal or C terminal of CLT1 and the constructs were expressed in *E. coli* BL21 at two different temperatures. 20 µl of bacterial whole cell extract (WCE) along with supernatants of low spin (LS; 5 min. at 5000g) and high spin (HS; 30 min. at 16000g) bacterial lysate were loaded on a SDS-PAGE. CGL2 was used as positive control for IPTG-induced expression and solubility. **B)** Likewise, 50 amino acids were truncated from either N-terminal or C terminal of CLT2 and the constructs were expressed in *E. coli* BL21 at 20°C. **C)** Bacterial growth effect of expressing the CLT2 truncated constructs were assessed in either IPTG induced (+) or non-induced *E. coli* BL21 cultures overnight at 20°C.

Table S1: Organisms used in this study

Name	Strain	Source/Reference
Caenorhabditis brenneri	PB2801	Caenorhabditis Genetics Center (CGC)
Caenorhabditis briggsae	AF16	Caenorhabditis Genetics Center (CGC)
Caenorhabditis elegans	N2	Caenorhabditis Genetics Center (CGC)
Caenorhabditis elegans	pmk-1	Caenorhabditis Genetics Center (CGC)
Caenorhabditis tropicalis	JU1373	Caenorhabditis Genetics Center (CGC)
Distolabrellus veechi	Environmental isolate	Luis Lugones, Utrecht University, Netherlands
Halicephalobus gingivalis	Environmental isolate	Pamela Fonderie, Ghent University, Belgium
Pristionchus pacificus	PS312	Iain Wilson, BOKU, Vienna, Austria
Aphelenchus avenae	Standard lab strain	Richard Sikora, University of Bonn, Germany
Aedes aegypti	Rockefeller	Pie Müller, Swiss Tropical and Public Health Institute, Basel, Switzerland
Coprinopsis cinerea	AmutBmut	(Swamy et al., 1984)
Botrytis cinerea	BC-3	Paul W. Sternberg, California Institute of Technology, Pasadena, USA
Escherichia coli	DH5α	
Escherichia coli	BL21(DE3)	Novagen
Escherichia coli	OP50	Michael Hengartner, University of Zürich, Switzerland

Table S2. Primers used in this study

Primer name	Sequence 5' - 3'
pF_CLT1	GGCGCATATGCCAGACACTTCCCTCGGATTCAAGATTATCAAAG
pR_CLT1	AGTGCGGCCGCTTAAGCTGAAGCATGCTGAAAG
pF_CLT2	CCCCATTAATGACGTTCGGTTCGCTCCGGTCTTCTTCCTCGAAT
pR_CLT2	GCTCGAGTGCGGCCGCTCACGCAGAGCCATCTTCC
pF_8His-CLT1	AATTCATATGCCACATCATCATCATCATCATCATCATGACACTTCCCTCGGATTCAAG
pR_8His-CLT1	AGTGCGGCCGCTTAAGCTGAAGCATGCTGAAAG
pF_8His-	CTGGTAGGCCCCTACTGTGGTGCATTC
pR_8His-	ATGATGATGATGATGATGATGATGTGGCATA
pF_8His-	TTAAGCGGCCGCACTCGAGCAC
pR_8His-	CTCGGCGAATGTGGCGCTCTCG
pF_8His-CLT2	CTTATTAATGACGCATCATCATCATCATCATCATCATTTCGGTTCGCTCCGGTCTTCTT
pR_8His-CLT2	GCTCGAGTGCGGCCGCTCACGCAGAGCCATCTT
pF_8His-	CATGTCACTGGTCATGCACTCGGCGGTGCC
pR_8His-	GGTGATGACCGTTGTAGTGGCCCTGGT
pF_8His-	ATCTTCCCCAAGGGCACTCCATT
pR_8His-	ATGATGATGATGATGATGATGATGCG
pF_8His-	TGAGCGGCCGCACTCGAGCACC
pR_8His-	GTCGACGCCGTGTTCTTCGCGG
pF_8His-	CTCTGGAAGAACAACGCCCAGACATTCGTCATC
pR_8His-	GTCGTAAGGCTGTGGCATCATCTCGATGG
pF_CLT1_qRT-	ATTTCGCCGCATTATACGACG
pR_CLT1_qRT-	AGGCTCGAAGGTGACTTCAG
pF_CLT2_qRT-	ACCTCTGGAAGAACAACTGCC
pR_CLT2_qRT-	TAACTTGGCGAGCTCCTTCG
pF_CGL2_qRT-	AGCATCACTGTCATCGACCA
pR_CGL2_qRT-	CCAGCGAGAATCCTAAGCAG
pF_Tubulin_qRT	CAATCCATCGCTCACCTCTC
pR_Tubulin_qRT	GCGTAATGTCTTGTCGATGTC

References:

Swamy, S., Uno, I., and Ishikawa, T. (1984). Morphogenetic Effects of Mutations at the A and B Incompatibility Factors in Coprinus cinereus. *Microbiology* 130**,** 3219-3224.
